# Supplementary material for: A Humanized Yeast Phenomic Model of Deoxycytidine Kinase to Predict Genetic Buffering of Nucleoside Analog Cytotoxicity
Source: Genes (Basel). 2019 Sep 30;10(10):770. doi: 10.3390/genes10100770 (PMC6826991; doi:10.3390/genes10100770)
Supplement: Supplementary file 1 [file genes-10-00770-s001.zip › Additional_Files/Additional_File7_GOTermHeatmaps.zip!/Additional_File7_GOTermHeatmaps/transcriptional_repressor_activity,_RNA_polymerase_II_transcription_regulatory_region_sequence-specific_binding.pdf]

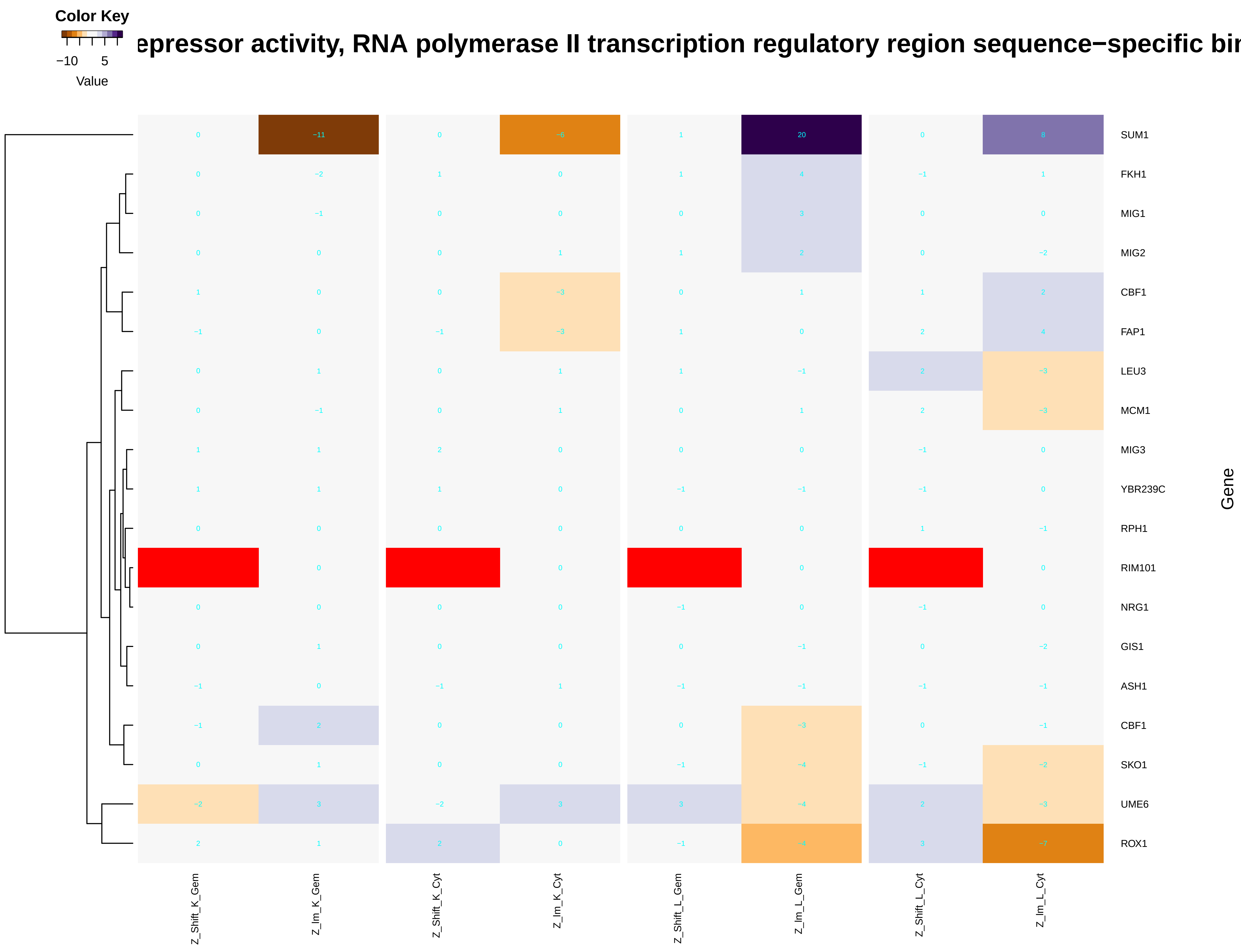
Color Key
−10 5
Value
epressor activity, RNA polymerase II transcription regulatory region sequence−specific bir
SUM1
FKH1
MIG1
MIG2
CBF1
FAP1
LEU3
MCM1
MIG3
YBR239C
RPH1
RIM101
NRG1
GIS1
ASH1
CBF1
SKO1
UME6
ROX1
Gene
Z_Shift_K_Gem
Z_Im_K_Gem
Z_Shift_K_Cyt
Z_Im_K_Cyt
Z_Shift_L_Gem
Z_Im_L_Gem
Z_Shift_L_Cyt
Z_Im_L_Cyt

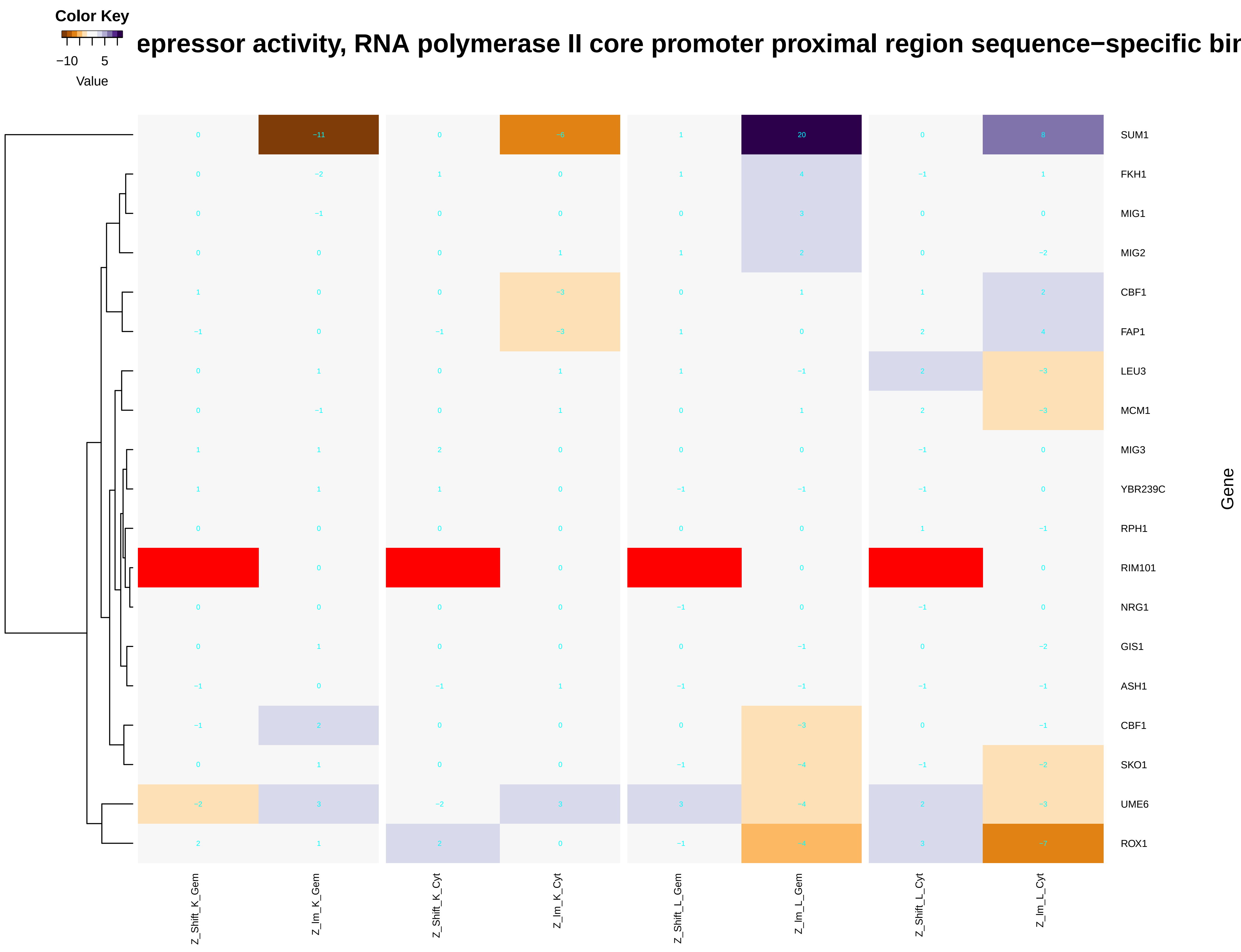
Color Key
−10 5
Value
epressor activity, RNA polymerase II core promoter proximal region sequence−specific bir
SUM1
FKH1
MIG1
MIG2
CBF1
FAP1
LEU3
MCM1
MIG3
YBR239C
RPH1
RIM101
NRG1
GIS1
ASH1
CBF1
SKO1
UME6
ROX1
Gene
Z_Shift_K_Gem
Z_Im_K_Gem
Z_Shift_K_Cyt
Z_Im_K_Cyt
Z_Shift_L_Gem
Z_Im_L_Gem
Z_Shift_L_Cyt
Z_Im_L_Cyt
